# Growth of *Cyanobacterium aponinum* influenced by increasing salt concentrations and temperature

**DOI:** 10.1007/s13205-014-0224-y

**Published:** 2014-05-18

**Authors:** Dominik Winckelmann, Franziska Bleeke, Peter Bergmann, Gerd Klöck

**Affiliations:** 1 School of Engineering and Science, Jacobs-University Bremen, Campus Ring 1, 28759 Bremen, Germany; 2 University of Applied Sciences Bremen, Am Neustadtswall 30, 28199 Bremen, Germany

**Keywords:** PAM, Bio fuel, *Cyanobacterium aponinum*, Evaporation, Open pond, Arid desert

## Abstract

The increasing requirement of food neutral biofuels demands the detection of alternative sources. The use of non-arable land and waste water streams is widely discussed in this regard. A Cyanobacterium was isolated on the area of a possible algae production side near a water treatment plant in the arid desert region al-Wusta. It was identified as *Cyanobacterium aponinum* PB1 and is a possible lipid source. To determine its suitability of a production process using this organism, a set of laboratory experiments were performed. Its growth behavior was examined in regard to high temperatures and increasing NaCl concentrations. A productivity of 0.1 g L^−1^ per day was measured at an alga density below 0.75 g L^−1^. *C. aponinum* PB1 showed no sign of altered growth behavior in media containing 70 g L^−1^ NaCl or less. Detection of a negative effect of NaCl on the growth using Pulse-Amplitude-Modulation chlorophyll fluorescence analysis was not more sensitive than optical density measurement.

## Introduction

Microalgae and cyanobacteria were considered to be a good choice as lipid source for bio diesel production, for CO_2_ sequestration, as nutritional supplement and for numerous other applications (Chisti 2008; Grobbelaar 2012). Recent studies consider microalgae and cyanobacteria to be not able to be used as a stand-alone commercial production process, but to be a means to utilize waste streams of other processes or to be a solution for a small-scale niche products (Van Beilen 2010; Chisti 2013). During the crude oil production in the Sultanate of Oman 800,000 m^3^ of contaminated production water accumulates per day (Breuer and Al-Asmi 2010). Part of the water is treated in a reed bed water treatment plant. Different options for further use of the treated water are under investigation. One possible option is to establish an algae growth facility for which the possible location would be in the arid desert region al-Wusta, Oman.

The use of non-arable land in a desert region has the advantage of sustainable production without competing with possible crop production (Singh et al. 2011), while it raises some possible difficulties. The maximum light exposure, annual temperature regime, cloud coverage, availability of water and the general infrastructure are considered to be major factors influencing the production in large-scale algae cultures (Grobbelaar 2009). Furthermore a high evaporation rate and the high consumption of fresh water to keep the salinity of the growth medium under a certain threshold is an important economic factor (Guieysse et al. 2013). The use of indigenous species as production organisms, either in axenic cultures or as mixed cultures, is said to circumvent some of the difficulties associated with the location (Mutanda et al. 2011; Odlare et al. 2011).

In preparation of the establishment of an algae-production site in an arid desert, the effect of warm temperature and increasing salt content on the growth of a domestic specie and the possible productivity were determined.

Therefore, the first step was to isolate different algae species from the water treatment plant. Thereby a strain of *Cyanobacterium aponinum* was found. *C. aponinum* is said to be able to grow in fresh and seawater media at temperatures of up to 45 °C (Moro et al. 2007) and might be used as lipid source (Karatay and Dönmez 2011).

The goal during the development of an algae growth process has to be to provide a process, which is able to deliver a stable biomass and product outcome, while being robust against outer circumstances. Grobbelaar (2000) gave an overview about the different fields and possibilities of improvement to optimize mass algal culture. He stated that process control should be improved to allow exploitation of physiological properties of the species to be grown. It was emphasized that carbon fixation and metabolism, and maximum photosynthetic rate are important to determine the overall efficiency of the photo capture and net carbon fixation, and possible damage to the photosystem reaction centers.

PAM chlorophyll fluorescence analysis were performed in laboratory studies to estimate CO_2_ fixation, light acclimation states (Behrenfeld et al. 2004; Campbell et al. 1998), and the influence of salt stress on photosynthesis (Lu and Vonshak 1999) in cyanobacteria and green algae using the pulse modulation principle (Schreiber et al. 1986). But the large-scale cultivation of algae opens up some new, so far not existing challenges and raise the need for real time online measurement methods (Havlik et al. 2013).

Chlorophyll fluorescence analysis was performed during this study using a phyto-PAM, which was designed to distinguish between fluorescence excited by light of four different preset wavelengths (Kolbowski and Schreiber 1995) and thereby enable an estimate of the relative pigment-content associated with those wavelengths.

## Methods

### Culture media

With regard to future industrial sized applications a simple media containing the commercially available fertilizer WUXAL^®^ Universaldünger liquid plant fertilizer (8 % N, 8 % P_2_O_5_, 6 % K2O, 0.01 % B, 0.004 % Cu, 0.02 % Fe, 0.012 % Mn, 0.004 % Zn; Wilhelm Haug GmbH & Co. KG, Germany) was used. The NaCl concentration added to the media was chosen corresponding to the salinity of the natural environment *C. aponinum* was isolated from. Wuxal liquid medium (WM) contained tap water with 0.002 % w/v MgSO_4_, 1 % w/v NaCl and 0.05 % v/v Wuxal. Wuxal was sterile filtrated and added after autoclaving.

## Isolation of algae

Environmental samples were taken in a reed bed system near Nimr, Sultanate of Oman (+18° 34′ 14.98″ N, +55° 49′ 5.99″ E). Initial growth was conducted in Erlenmeyer flasks containing WM soil media (WM with 3 % v/v soil extract (Watanabe 2005)) for 7 days at room temperature and natural light. After biomass increase was visible, the flasks were transferred into a light incubator (RUMED Licht Thermostat Typ 1301, Rubarth Apparate GmbH, Germany) and cultivated at 40° C and 110 ± 2.5 PAR m^−2^ s^−1^. The flasks were shaken constantly at 150 rounds per minute (orbital shaker 3005, GFL—Gesellschaft für Labortechnik mbH, Germany). Single-celled algae were isolated using the 13-streak method on WM- soil plates containing 1.5 % (w/v) Agar. The agar plates were grown in the light incubator until growth was visible. Colonies containing only one algae species were transferred to liquid media.

### Identification of algae

Genomic DNA was extracted from freshly grown *C. aponinum* PB1. Cells were broken-up via beat mill and DNA was extracted using a DNA extraction kit (DNeasy Plant Mini kit, Qiagen, Germany).The 16 s rRNA gene was amplified by PCR using the primers CYA106F, and a mixture of CYA781R (a) and CYA781R (b) (Boutte et al. 2006); (GenBank: JN584264.1)) and the internal transcribed spacer between the 16S and 23S rRNA genes (ITS) was amplified using the primer pair ULR and CSIF (without the G-C clamp (Janse et al. 2003); GenBank: KF982001). The PCR product was purified using the QIAquick PCR Purification Kit (Qiagen, Germany). Sequencing was performed using the Sanger sequencing technique by GATC Biotech AG (Germany).

### Characterization of algae

The optical density was measured at 700 nm (OD700) using a Thermo Spectronic Genesys 20 Model 4001/4 (Thermo Electron Corporation, USA). For the determination of bio dry weight, part of a culture was washed and filtered. The filter was dried at 105 °C until the weight was constant.

Fluorescence analysis was performed using a phyto-PAM with phyto-EDF (Walz, Germany). The samples were transferred in a 1.5 ml cup and the perspex-rod sensor tip was 0.2 mm immersed into the algae suspension. Everything was covered and the fluorescence was measured (Settings: gain was adjusted to F 645 nm values above 200 and below 400, measuring light intensity 0, saturation light intensity 10, saturation pulse length 400 ms; For introduction, see (Schreiber et al. 1995)).

Each chlorophyll fluorescence measurement performed delivered four light-adapted steady state fluorescence values (*F*′*x*) and four maximum fluorescence values (*F*′*mx*), which were assigned to one of the four different used excitation wave length (*x* = 470, 520, 645 or 665 nm). The effective quantum efficiency of charge separation at photosystem II (yield) in actinic light was calculated according to Genty et al. (Kaplan et al. 1989) for each wavelength used (yield *x*).

$$\text{Yield}=\frac{F^{\prime }m\;\text{-}\;F}{F^{\prime }m}$$

To determine a shift in light utilization the ratio of steady-state fluorescence was calculated. The maximum signal of *F*′*x* was set as 1, the lower values were normalized against it resulting in three values called rel.*Fx*.

### Microalgae cultivation

Stock cultures of *C. aponinum* PB1 were grown in Erlenmeyer flasks at 24 ± 2 °C and illuminated with white fluorescent light (60 ± 2.5 PAR m^−2^ s^−1^, measured using Meteon Irradiance meter with a PAR Lite sensor (Kipp and Zonen, Netherlands)). Temperature was measured using a Hobo^®^ Pendant temp #UA-001-64 (Onset Computer Corporation, USA).

The growth experiment was performed at 38 ± 2 °C. All cultures were illuminated with white fluorescent light (110 ± 2.5 PAR m^−2^ s^−1^ for 14 h per day, in 49 cm high glass tubes with an inner diameter of 49 mm and a total liquid volume of 750 ml, and aerated with fresh air at a rate of 100 l/h. Initial inoculum was 1 × 10^6^ cells per ml. The OD700, cell concentration (determined using a Zeiss Axiostar Plus light microscope (Carl Zeiss, Germany) and a Thoma Neu chamber with a depth of 0.1 mm (Paul Marienfeld GmbH & Co. KG, Germany), pH (using a wtw pH 315i (WTW Wissenschaftlich-Technische Werkstätten GmbH, Germany)) with an InLab Micro pH electrode (Mettler Toledo GmbH, Germany)), and chlorophyll fluorescence were determined twice a week before nine in the morning. Evaporated water was replaced with tap water or a NaCl solution (10 % w/v NaCl in tap water) and salinity was measured twice a week (using a conductivity meter LF 340 with a TetraCon^®^ 325 (WTW Wissenschaftlich-Technische Werkstätten GmbH, Germany).

## Results

### Characterization of *C. aponinum* PB1

The sequence for the 16S rRNA gene covered 412 bases and the sequence gained for the ITS region covered 441 bases. After BLAST (Altschup et al. 1990) analysis a similarity of 99 % with an *E* value of 0.0 to *C. aponinum* PCC 10605 (GenBank: CP003947.1), respectively, 100 % and *E* value of 0.0 for the 16S rRNA gene alone (NR_102443.1), and a similarity of 98 % with an *E* value of 0.0 to *C. aponinum* ETS-03 (GenBank: AM238427.1) were found.

**Fig. 1:**
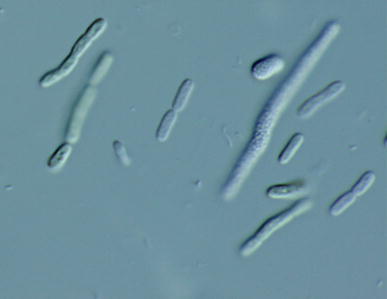
Picture of *C. aponinum* PB1 (Culture was grown for 14 days in WM), Picture was made by Gerhard Kauer with light microscope and 630-fold magnification

*Cyanobacterium aponinum* PB1 grow in peanut-like or cylindrical shape with a length of 3–4 µm and a diameter of 2–3 µm. They appear solitary or during cell division as pairs. Grown under nutrient-limited conditions they show elongated cell shapes (Fig. 1). They appear blue–green in color.

**Fig. 2:**
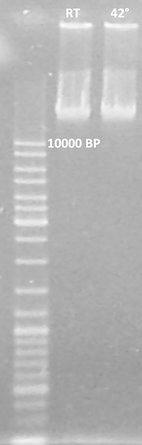
No indication of *C. aponinum* genomic DNA fragmentation after 2 h incubation at 42° C (*right lane*) compared to 2 h incubation at room temperature (RT, *left lane*). DNA fragmentation was performed like described in Nedelcu 2006

*Cyanobacterium aponinum* PB1 is able to grow at 45 °C and 110 PAR m^−2^ s^−1^ (14 h light and 10 h dark) in WM with 10 g L^−1^ NaCl. Grown using the same experimental set-up at 28 °C and 180 PAR m^−2^ s^−1^ (14 h light and 10 h dark) exponential growth was achieved over a period of 14 days with a growth rate *µ* per day of 0.3 (SD 0.006) and a maximum light-adapted yield 645 nm of 0.45 (data not shown). The constant bio dry weight of washed cultures with an OD700 of 1 was 0.51 g L^−1^ (SD 0.0075). Cultures grown at RT showed no DNA fragmentation (Nedelcu 2006) after incubation at 42 °C in the dark (Fig. 2).

### Growth in WM with increasing NaCl content

**Fig. 3:**
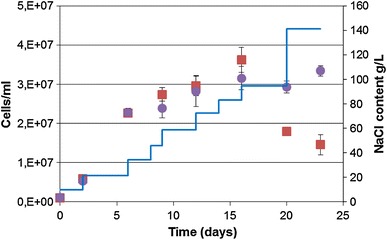
Growth of *C. aponinum PB1* in WM with increasing NaCl content (filled squares; NaCl content as line) over time. As control a *C. aponinum* PB1 culture growing in WM with 10 g L^−1^ NaCl is shown (*filled circles*). *Error bars* represent three times the standard deviation

**Fig. 4:**
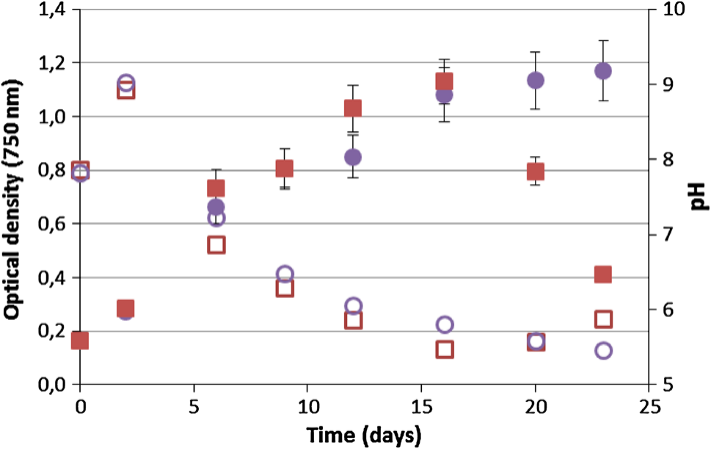
pH change and growth as measured by optical density of *C. aponinum PB1* with increasing NaCl content (pH: *open squares*; OD: *filled squares*) over time. As control a *C. aponinum* PB1 culture growing in WM with 10 g L^−1^ NaCl is shown (pH: *open circles*; OD: *filled circles*). *Error bars* represent three times the standard deviation

*Cyanobacterium aponinum* PB1 was grown in media with increasing NaCl concentrations (referred to as treated culture), namely 10, 13, 20, 26, 35, 58, 70, 92, and 140 g L^−1^, and stable NaCL concentration (10 g L^−1^, referred to as control culture). Growth was exponential for both cultures till day 6, when the NaCl content was increased to 20 g L^−1^, and their growth rate *µ* per day was comparable with 0.35 for the treated culture and 0.33 for the control. Afterwards, the growth was linear with an increase of 1.3 × 10^6^ cells per ml per day for the treated and 0.9 × 10^6^ cells per ml per day for the control culture till day 16, after which the NaCl concentration was increased to 92 g L^−1^. The cell density of treated culture decreased from 3 × 10^7^ to 1.8 × 10^7^ cells/ml during the growth in WM containing 92 g L^−1^ NaCl and even further to 1.5 × 10^7^ cells/ml after the NaCl content was increased to 140 g L^−1^. The cell content in the control culture stayed stable at 3 × 10^7^ cells per ml over the whole time (Fig. 3). The optical density showed a similar course like the cell count (Fig. 4). The coloration became less green and paler after the NaCl content was 92 g L^−1^ or higher. The pH rose in the first 2 days from 7.8 to 9 on day 6, when the NACL content was increased to 20 g L^−1^, but decreased till day 16, after which the NaCl concentration was increased to 92 g L^−1^, to 5.8 for the treated culture and 5.5 for the control. Over the whole course the pH of the treated culture was slightly higher. This changed after the NaCl content was increased to 92 g L^−1^ when the pH of both cultures was at 5.6. The pH of the treated culture stayed stable during growth in 140 g L^−1^ Nacl while the pH of the control culture slightly increased (Fig. 4).

**Fig. 5:**
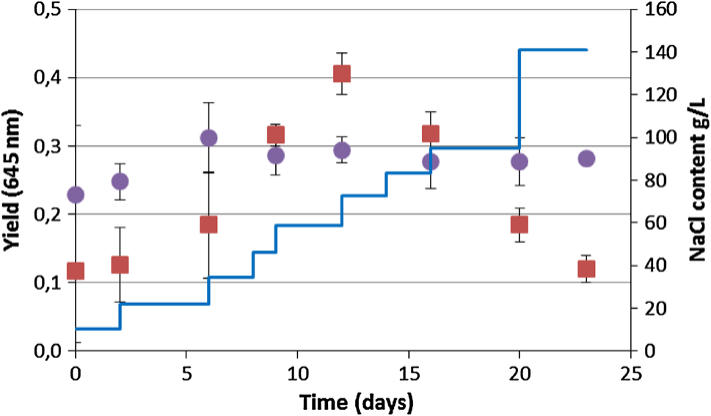
Photosynthetic yield of *C. aponinum PB1* with increasing NaCl content (*filled squares*; NaCl content as *line*) over time. As control a *C. aponinum* PB1 culture growing in WM with 10 g L^−1^ NaCl is shown (*filled circles*). *Error bars* represent three times the standard deviation

The yield of the treated culture increased from 0.12 in media containing 10 g L^−1^ NaCl to 0.4 in media containing 58 g L^−1^ NaCl and decreased stepwise to 0.12 when the culture was grown in media containing 140 g L^−1^ NaCl. The yield of the control culture increased from 0.22 to 0.31 and stabilized at 0.28 (Fig. 5).

**Fig. 6:**
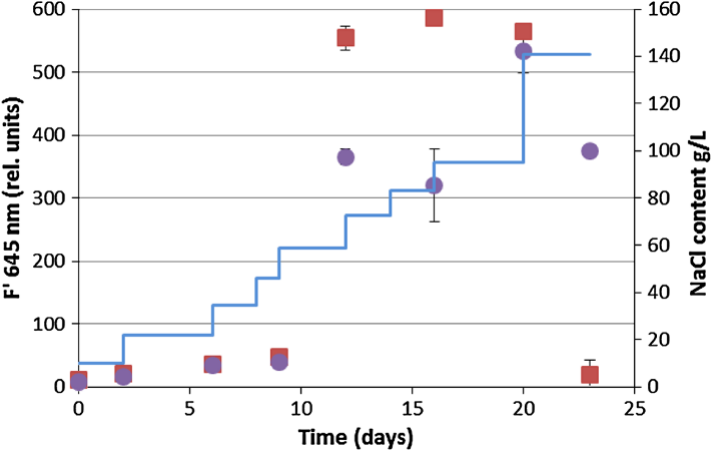
Change of light-adapted steady state fluorescence (*F*′) of *C. aponinum PB1* with increasing NaCl content (*filled squares*; NaCl content as *line*) over time. As control a *C. aponinum* PB1 culture growing in WM with 10 g L^−1^ NaCl is shown (*filled circles*). *Error bars* represent three times the standard deviation

The light-adapted fluorescence (F′ = 645 nm) of the treated and the control culture increases from 10 at a NaCl content of 10 g L^−1^ to 50 rel. units after growth in media containing 35 g L^−1^ NaCl. The fluorescence values increase steeply during the growth in WM with 58 g L^−1^ NaCl for both cultures, but while it stays stable for the treated culture till the NaCl content was increased to 140 g L^−1^ NaCl, after which the fluorescence values drop to undetectable values, it decreases for the control culture on day 16, increases till day 20 and declines till day 23 (Fig. 6).

The relative, normalized fluorescence values of the control culture stayed stable from day 6 to 16, after which the NaCl concentration was increased to 92 g L^−1^. Thereafter, RelF 520 nm and RelF 665 nm increased slightly. The relF 520 nm of the stressed culture was slightly higher than the control value and decreased after the NaCl content was increased to 58 g L^−1^. RelF 665 nm of the stressed culture showed similar values like the control culture declined after day 10.

## Discussion

The isolate was assigned to be *C. aponinum* because of the molecular similarities, similar shape and color, and the ability to survive in BG11 media containing 34 g L^−1^ salt. All those features are used to distinguish *C. aponinum* ETS-03 from other Cyanobacteria and to establish the *C. aponinum* species (Moro et al. 2007). Our isolate was found in microbial mats in a warm water pond and able to be free living like *C. aponinum* ETS-03 (Moro et al. 2007). *C. aponinum* PB1 showed a change in color during temperature shift or nutrient stress (data not shown) and Moro reported unknown carotenoids in the *C. aponinum* ETS-03. *C. aponinum* was found to accumulate up to 45 % lipids per dry weight (Karatay and Dönmez 2011). Because of the ability to most likely survive the temperature conditions to be found at the test field area and the prospect of possible products made us chose *C. aponinum* PB1 for further tests in the laboratory.

*Cyanobacterium aponinum* PB1 showed no difference in growth until day 8 when the NaCl content was increased to 92 g L^−1^. The control had reached the stationary phase at that time, while the other culture declined in OD and in cell concentration. *C. aponinum* PB1 could be part of the functional group of moderately halotolerant cyanobacteria, as defined by Reed and Stewart ((Reed and Stewart 1985). This group is able to synthesize Glucosylglycerol [O-a-D-glucopyranosyl-(1– >2)-glycerol] as their major compatible solute as response to increasing salt concentrations. Members of this group were found in sea and fresh water and were able to survive in media containing salt concentrations of double the amount of salt in sea water and even more (Reed and Stewart 1985). Hagemann defined the NaCl tolerance limit of moderately halotolerant strains as 1.7 M, which coincides with our own findings (1.7 M NaCl ~99 g L^−1^).

It was shown that cyanobacteria alkalize the growth media when not provided with inorganic carbon (IC) (Kaplan et al. 1989). Cyanobacteria store CO_2_ in form of IC in carboxysomes (Reinhold et al. 1991). The initial increase of pH during this experiment indicates an insufficient internal IC pool. This pool is filled over time by the CO_2_-concentrating mechanism (Kupriyanova et al. 2013) using the excess CO_2_ from the air leading to a decrease in pH. *C. aponinum* PB1 alkalized the medium to pH values of up to 10.5 during experiments using an insufficient aeration rate (data not shown). Cyanobacteria are able to survive salt stress by a number of different physiological changes. The decrease of pH until day 20 of the culture grown in WM with increasing NaCl compared to the control culture could be because of an increase in Na^+^/H^+^-antiporter synthesis during growth in elevated salt levels (Allakhverdiev et al. 1999). It was shown that the artificial induction of a Na^+^/H^+^-antiporter overexpression enabled a freshwater cyanobacterium to survive and grow in seawater (Waditee et al. 2002). Another response to increased salt levels is an increase in unsaturation of membrane lipids leads to a change in plasma membrane mobility (Allakhverdiev et al. 2000a). This might depress the functionality of K^+^ channels, which are also point of entry for Na^+^ molecules and of water channels (Allakhverdiev et al. 2000b). While in the same moment the synthesis of membrane bound Na^+^/H^+^-antiporter and H^+^ATPase-enzymes might be increased (Allakhverdiev et al. 2001). All those processes are thought to decrease the Na^+^ content in the cytosol and thereby protecting the photosystems against salt-induced inactivation (Singh et al. 2002). The counteracting mechanisms of increased number of enzymes and depression of functionality limit the survival under stress to a certain threshold, which is thought to be reached by the culture growing in increasing salt levels on day 20, like it is shown in change in pH, growth rate and photosynthetic activity. The mechanisms of lipid unsaturation and solute biosynthesis are said to be not only responsible for salt tolerance but also for tolerance to osmotic or high and low temperatures (Klähn and Hagemann 2011; Singh et al. 2002). Modern proteomic analyses try to explain the mechanisms of heat shock and salt-induced stress in more detail (Castielli et al. 2009; Ludwig 2012).

The photosynthetic yield 645 nm was below the maximum yield of the stock cultures grown at room temperature. The cultures photosynthetic capability was limited over the whole course of the experiment. The highest yield 645 nm was measured after growth in media with 58 g L^−1^ with 0.4. At the same day, the OD was significantly higher compared to the control culture. Afterwards, the yield decreased steadily and a decrease in growth could not be seen before a decrease in cell concentration or OD was measurable. Also the other fluorescence parameters were not reliable to indicate NaCl stress. Under controlled laboratory conditions, fluorescence values of dark-adapted and biomass-standardized samples were found to be reliable indicators for various stressors (Lu and Vonshak 1999). But the use of fluorescence values under less controlled conditions was found to be not reliable (Kruskopf and Flynn 2006).

The utilization of pH measurements as control parameter for culture stability could be used to indicate CO_2_ depletion. A different method or changes in the measurement procedure have to be found to use PAM as control parameter for culture stability. A bypass with rest time for dark adaption might be an answer for semi-continuous online measurement.

The possible growth in temperatures above 30 °C in easy-made liquid media in combination with its free-living growth form predestine *C. aponinum* PB1 for field tests in the Omanian desert.

The mean conductivity of the feed water was measured with 14 ms cm^−1^ (personal communication). The maximum natural evaporation on the Arabic peninsula is difficult to measure and values of up to 14 mm per day are reported (Alsharhan et al. 2001). Evaporation would be even higher because of the by circulation enlarged surface of the algae culture. We assumed a maximum evaporation of 20 mm per day. The theoretical maximal conductivity, while harvesting every third day, due to evaporation would be 24 ms cm^−1^, which correlates to the conductivity of a NaCl solution with 14 g L^−1^ (Lewis 1980). Therefore, as long as the main salt in the feed water is NaCl (Nübel et al. 2000) *C. aponinum* PB1 should be able to grow. The salt in the feed water might have a positive impact on the lipid content because higher salt levels were shown to increase lipid accumulation in cyanobacteria (Araujo et al. 2011; Bhadauriya et al. 2008).

Assuming a theoretical exponential growth with an average growth rate of 0.3 µ per day in a 10 cm deep open pond a biomass yield of 22,500 kg per hectare and year (expecting 300 working days) could be achieved. With a caloric value of 20 kJ per 10 g dry weight, like it was published for other cyanobacteria (Quintana et al. 2011), this would correspond to an energy content of 50,000 Mj. Similar and higher growth rates were reported for other cyanobacteria (Markou and Georgakakis 2011). A possible lipid yield of up to 10,125 kg of lipids per hectare and year if 45 % lipids per dry weight might be achieved (Karatay and Dönmez 2011). The usability as biodiesel highly depends on the lipid composition (Quintana et al. 2011), which is influenced by the growth stage, growth temperature and light intensity (Liu et al. 2005; Vargas et al. 1998; Walsh et al. 1997).

Presume exponential growth at dry weight concentrations below 0.37 g L^−1^ and harvesting two-third of the whole volume every three days 65,000 m^3^ have to be moved per year. Assuming an average evaporation rate of 14 mm per day additional 140,000 m^3^ of water have to be pumped. This accumulates to an energy consumption of 20,000 Mj for liquid movement alone.

To be able to give a final statement about the economic feasibility of an algae production process using *C. aponinum* the actual biomass composition achievable in large scale and possible synergies with established procedures have to be evaluated (Patel et al. 2012). 
